# Polymorphism in GRHL2 gene may contribute to noise-induced hearing loss susceptibility: a meta-analysis^☆^

**DOI:** 10.1016/j.bjorl.2019.01.003

**Published:** 2019-02-23

**Authors:** Xin Li, Zhengping Zhu, Wei Li, Li Wei, Baocheng Zhao, Zheng Hao

**Affiliations:** a Nanjing Municipal Center for Disease Control and Prevention, Department of HIV/AIDS/STI Prevention and Control, Jiangsu, China; b Southeast University, School of Public Health, Department of Epidemiology and Health Statistics, Key Laboratory of Environmental Medicine Engineering, Jiangsu, China; c Nanjing Municipal Center for Disease Control and Prevention, Environmental Health Division, Jiangsu, China; d Nanjing Zhongyangmen Community Health Service Center, Kang’ai Hospital, Center of Diagnosis and Treatment for Developmental Dysplasia of the Hip, Jiangsu, China

**Keywords:** Noise-induced hearing loss, Grainyhead like 2, Molecular epidemiology, Polymorphism, Meta-analysis, Perda auditiva induzida por ruído, Grainyhead like 2, Epidemiologia molecular, Polimorfismo, Metanálise

## Abstract

**Instruction:**

Noise-induced hearing loss is a leading occupational disease caused by gene-environment interaction. The Grainy Like 2, GRHL2, is a candidate gene. In this regard, many studies have evaluated the association between GRHL2 and noise-induced hearing loss, although the results are ambiguous and conflicting.

**Objective:**

The purpose of this study was to identify a precise estimation of the association between rs3735715 polymorphism in GRHL2 gene and susceptibility of noise-induced hearing loss.

**Methods:**

A comprehensive search was performed to collect data up to July 8, 2018. Finally, 4 eligible articles were included in this meta-analysis comprising 2410 subjects. The pooled odds ratios with 95% confidence intervals were used to evaluate the strength of the association.

**Results:**

Significant association was found in the overall population in the dominant model (GA/AA vs. GG, odds ratio = 0.707, 95% confidence interval = 0.594–0.841) and allele model (G allele vs. A allele, odds ratio = 1.189, 95% confidence interval = 1.062–1.333). When stratified by source of the subjects, we also found association between rs3735715 and noise-induced hearing loss risk in the dominant model (GA/AA vs. GG, odds ratio = 0.634, 95% confidence interval = 0.514–0.783) and allele model (G allele vs. A allele, odds ratio = 1.206, 95% confidence interval = 1.054–1.379).

**Conclusion:**

Rs3735715 polymorphism in GRHL2 gene may influence the susceptibility of noise-induced hearing loss. Additional large, well-designed and functional studies are needed to confirm this association in different populations.

## Resumo

**Introdução:**

Perda auditiva induzida por ruído é uma das principais doenças ocupacionais causadas pela interação gene-ambiente. O *Grainy Like 2*, ou *GRHL2* é um gene que tem sido considerado como candidato. Nesse sentido, muitos estudos avaliaram a associação entre o *GRHL2* e perda auditiva induzida por ruído, embora os resultados sejam ambíguos e conflitantes.

**Objetivo:**

Identificar uma estimativa precisa da associação entre o polimorfismo rs3735715 no gene *GRHL2* e a suscetibilidade à perda auditiva induzida por ruído.

**Método:**

Uma pesquisa abrangente foi feita para coletar dados até 8 de julho de 2018. No fim, quatro artigos elegíveis foram incluídos nesta metanálise, abrangeram 2.410 indivíduos. As *odds ratios* agrupadas com intervalos de confiança de 95% foram usadas para avaliar a força da associação.

**Resultados:**

Uma associação significante foi encontrada na população geral no modelo de dominância (GA/AA vs. GG, *odds ratio* = 0,707, intervalo de confiança 95% = 0,594-0,841) e modelo de alelo (alelo G vs. alelo A; *odds ratio* = 1,189, intervalo de confiança 95% = 1,062 a 1,333). Quando estratificados pelo local de trabalho dos indivíduos, também encontramos associação entre rs3735715 e risco de perda auditiva induzida por ruído no modelo de dominância (GA/AA vs. GG, *odds ratio* = 0,634, intervalo de confiança 95% = 0,514 ± 0,783) e modelo de alelo (alelo G vs. alelo A; *odds ratio* = 1,206, intervalo de confiança 95% = 1,054- 1,379).

**Conclusão:**

O polimorfismo Rs3735715 no gene *GRHL2* pode influenciar a suscetibilidade à perda auditiva induzida por ruído. Estudos adicionais, amplos, bem desenhados e funcionais são necessários para confirmar essa associação em diferentes populações.

## Introduction

Noise-induced hearing loss (NIHL) is one of the worldwide leading occupational disease especially in the developing countries and the second most frequent form of sensorineural hearing deficit after Age-Related Hearing Impairment (ARHI).^1^ It is a complex hearing impairment induced by a combination of genetic and environment factors.^2^ Recently, accumulating epidemiological evidence indicated that noise, organic solvents, heat, heavy metals, vibrations, smoking, drinking, high blood pressure and cholesterol levels are responsible environmental factors.^2^, ^3^, ^4^, ^5^, ^6^, ^7^, ^8^ Additionally, it was demonstrated that genetics contribute to the incidence of NIHL, deduced from animal experiments.^9^, ^10^ Some of these studies used heterozygote or homozygote knockout mice model and confirmed that otocadherin 23 (cdh23) gene,^11^ glutamate peroxidase 1 (gpx1) gene,^12^ plasma membrane Ca^2+^-ATPase isoform 2 (pmca2) gene,^13^ and heat shock factor (hsf1) gene^14^ might associate with NIHL risk. In human, several association studies have demonstrated that CDH23 gene,^15^, ^16^ human 8-oxoG DNA glycosylase 1 (hOGG1) gene,^17^ catalase (CAT) gene,^18^ heat shock protein 70 (HSP70) gene,^19^ potassium voltage-gated channel, Isk-related family, member 1 (KCNE1) gene and potassium voltage-gated channel, KQT-like subfamily, member 4 (KCNQ4) gene^20^ might be involved in the susceptibility to NIHL.

The Grainy Like 2 (GRHL2) is a transcription factor which is associated with the composition of the organ of Corti.^21^ Grainyhead-like transcription factor family includes three members (GRHL1–GRHL3) regulating epithelial adhesion.^22^ GRHL2 is highly expressed in cochlear duct lining cells and plays an important role in the epithelial cell maintenance and embryonic development.^23^ GRHL2 gene knockout mice were embryonically lethal.^22^ Recently, two research teams confirmed that GRHL2 gene might influence the susceptibility to ARHI and progressive autosomal dominant hearing loss (DFNA28).^22^, ^24^ Up to now, promising but contradictory data showed that the GRHL2 gene might be responsible for the development of NIHL. GRHL2 gene is located on chromosome 8q22.3, including 15 introns and 16 exons. Currently, several candidate gene studies have focused on whether the GRHL2 gene is associated with NIHL risk while the results remain conflicting rather than conclusion. Yang et al. genotyped the potentially functional polymorphism rs3735715 and got a significant association.^25^ Xu et al. confirmed this founding in another population.^26^ But Li et al. did not find any association between rs3735715 and NIHL susceptibility.^27^ In this study, we performed a meta-analysis to estimate the overall association.

## Methods

### Search strategy and date extraction

We searched all literatures from PubMed, CNKI, Wang Fang, Web of Science and Springer databases, using the keyword (*GRHL2* or “Grainyhead Like 2” or rs3735715) and (NIHL or “Noise-Induced Hearing Loss”). The search time was not limited. The last search update was done on July 8^th^, 2018. Articles investigating GRHL2 and NIHL before July 8^th^, 2018 were all included in this meta-analysis. The first study was in 2013. Four literatures were included in our meta-analysis according to the following criteria: (1) was a case-control study; (2) was a study about the GRHL2 polymorphism and NIHL susceptibility; (3) with usable data for allele frequency; (4) was a paper written in English or Chinese. Two of the authors extracted the available data independently according to the criteria mentioned above. We extracted the information including publication year, the name of the first author, country, ethnicity, source of the subjects, and genotype distributions of the GRHL2 rs3735715 polymorphisms among cases and controls. The controversies were discussed within our research team, and we reached a consensus eventually.

### Statistical methods

Summary Odds Ratios (ORs) with 95% Confidence Intervals (CIs) determined by *Z*-test were used to assess the strength of the association. If the *p*-value was less than 0.05, the association was considered significant. Stratified analysis was performed by source of the subjects. We used *Q* test to assess the between-study heterogeneity. The heterogeneity was considered to be significant if the *p*-value was less than 0.10. *I*^2^ statistic (*I*^2^ = 100% × (*Q* − df)/*Q*) was also used to quantify heterogeneity. *I*^2^ greater than 50% indicated heterogeneity among studies. The fixed-effects model and random-effects model were used to pool the data appropriately.^28^ The fixed-effects model was used when there was no heterogeneity existed. It assumes that all the studies are sampled from the populations with same effect size. The fixed-effects model makes an adjustment to study weights according to the in-study variance. The random-effects model based on the Dersimonian and Laird method was more suitable when the heterogeneity existed; otherwise the two methods provided the same results.

To test for publication bias in this meta-analysis, we performed both Egger's and Begg's test.^29^ The publication bias was assessed by funnel plot and the linear regression asymmetry test.

All analyses were performed using Stata software version 8.2 (Stata Corporation, College Station, TX, USA).

## Results

### The characteristics of the included studies

**Figure 1:**
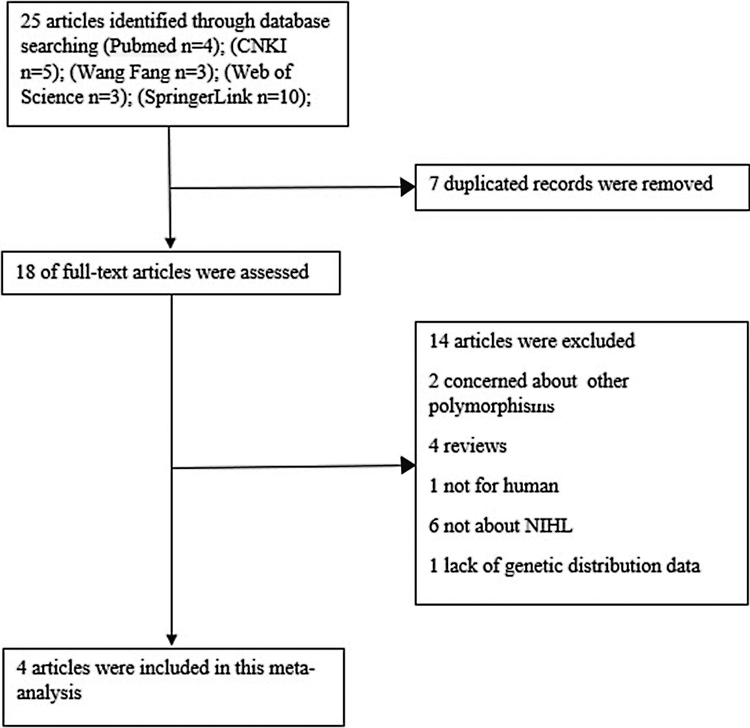
Flow diagram of study inclusion/exclusion.

A total of 25 relevant studies were identified through database screening. Seven were excluded for duplicated records. After a detailed evaluation of the full-text of the 18 studies, 14 were excluded: two concerned about other polymorphisms, 4 reviews, 1 not for human, 6 not about NIHL and one lack of genetic distribution data. 4 articles were eventually included in the analysis. The flow of studies through this meta-analysis was shown in Fig. 1. Characteristics of the 4 studies and details of the genotype distributions were shown in Table 1, Table 2.

**Table 1:** Basic information of the 4 studies in this meta-analysis.

Year	First author	Country	Ethnicity	Source of the subjects	Cases	Controls
2018	Yang	China	Asian	Steel factory	340	339
2016	Yang	China	Asian	Steel factory	283	281
2013	Li	China	Asian	Chemical fiber company	340	356
2016	Xu	China	Asian	Steel factory	236	235

**Table 2:** Genotype distributions of the GRHL2 rs3735715 polymorphisms among cases and controls.

Year	First author	Cases			Controls		
		GG	GA	AA	GG	GA	AA
2018	Yang	115	157	68	85	181	73
2016	Yang	94	126	63	67	154	60
2013	Li	122	161	57	119	162	75
2016	Xu	80	104	52	56	130	49

### The demographics of the subjects of the 4 studies in this meta-analysis

The average of age in cases was 40.7 ± 8.4, 40.5 ± 8.1, 39.3 ± 5.8, and 40.4 ± 8.3 for Yang's (2018), Yang's (2016), Li's (2013), and Xu's (2016) study, respectively. The average of age in controls was 40.0 ± 8.4, 39.8 ± 8.1, 39.8 ± 5.8, and 39.5 ± 8.2 for Yang's (2018), Yang's (2016), Li's (2013), and Xu's (2016) study, respectively. The hearing threshold level in cases was 51.0 ± 9.0, 37.6 ± 11.7, and 51.4 ± 8.8 for Yang's (2016), Li's (2013), and Xu's (2016) study, respectively. The hearing threshold level in controls was 11.7 ± 10.7, 14.2 ± 3.9, and 9.3 ± 9.1 for Yang's (2016), Li's (2013), and Xu's (2016) study, respectively. The demographics of the subjects of the 4 studies in this meta-analysis were detailed in Table 3.

**Table 3:** The demographics of the subjects of the 4 studies in this meta-analysis.

First author (year)	Age (years) (mean ± SD)		Gender		Hearing threshold level (dB) (mean ± SD)		Time of exposition to the noise (years) (mean ± SD)	
	Cases	Controls	Cases	Controls	Cases	Controls	Cases	Controls
Yang (2018)	40.7 ± 8.4	40.0 ± 8.4	326 male	326 male	NA	NA	NA	NA
			17 female	17 female				
Yang (2016)	40.5 ± 8.1	39.8 ± 8.1	274 male	274 male	51.0 ± 9.0	11.7 ± 10.7	18.9 ± 9.1	18.3 ± 8.8
			12 female	12 female				
Li (2013)	39.3 ± 5.8	39.8 ± 5.8	306 male	317 male	37.6 ± 11.7	14.2 ± 3.9	17.0 ± 6.9	17.0 ± 7.0
			34 female	39 female				
Xu (2016)	40.4 ± 8.3	39.5 ± 8.2	239 male	239 male	51.4 ± 8.8	9.3 ± 9.1	18.7 ± 9.2	18.7 ± 9.2
			0 female	0 female				

NA, data not available.

### Overall population

Overall, our meta-analysis showed a significant association between rs3735715 and NIHL risk in both dominant model and allele model. For dominant model (GA/AA vs. GG) OR = 0.707, 95% CI = 0.594–0.841. For allele model (G allele vs. A allele) OR = 1.189, 95% CI = 1.062–1.333 (Table 4).

**Table 4:** Meta-analysis of the GRHL2 rs3735715 polymorphism and NIHL risk.

Population	Comparison	Test of association	*p*^a^	Test of heterogeneity	
		OR (95%CI)		*p*^b^	*I*^2^ (%)
Overall	GA/AA vs. GG	0.707 (0.594–0.841)	<0.001	0.343	10
	AA vs. GA/GG	0.928 (0.761–1.130)	0.455	0.584	0
	G allele vs. A allele	1.189 (1.062–1.333)	0.003	0.977	0
Source of the subjects steel factory	GA/AA vs. GG	0.634 (0.514–0.783)	<0.001	0.964	0
	AA vs. GA/GG	1.001 (0.794–1.261)	0.993	0.815	0
	G allele vs. A allele	1.206 (1.054–1.379)	0.006	0.971	0
Chemical fiber company	GA/AA vs. GG	0.897 (0.656–1.226)	0.496	–	–
	AA vs. GA/GG	0.755 (0.515–1.106)	0.149	–	–
	G allele vs. A allele	1.149 (0.928–1.421)	0.202	–	–

a *p*-value determined by *Z* test.

b *p*-value determined by *Q*-test.

### Subgroup analysis by source of the subjects

Stratification by source of the subjects identified a significant association in steel factory population between GRHL2 rs3735715 polymorphism and NIHL risk. Among the subjects chosen from steel factory, significant association was found in the dominant model (GA/AA vs. GG) OR = 0.634, 95% CI = 0.514–0.783, and allele model (G allele vs. A allele) OR = 1.206, 95% CI = 1.054–1.379 (Table 4). We did not find any significant association between the GRHL2 rs3735715 polymorphism and NIHL risk in the chemical fiber company workers.

### Heterogeneity and publication bias

The meta-analysis was performed using a fixed-effects model based on the Mantel-Haenszel method because the between-study heterogeneity was not found (Table 4).

**Figure 2:**
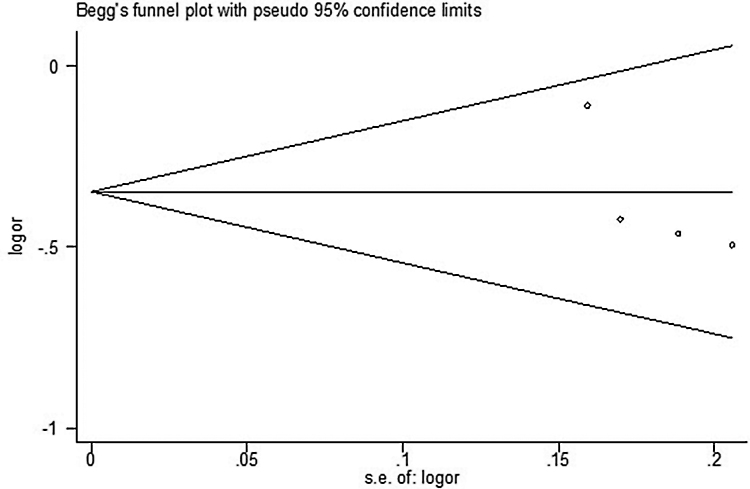
Begg's funnel plot for publication bias test using the dominant model (GA/AA vs GG).

Publication bias is always a concern in a meta-analysis. As shown in Fig. 2, there was no obvious asymmetry in the shape of the funnel. We also performed the Egger's test to evaluate the funnel plot symmetry. The results showed no evidence of the publication bias (*t* = −1.92, *p* = 0.194 for GRHL2 rs3735715 GA/GG vs. GG).

## Discussion

In the present study, the GRHL2 rs3735715G > A polymorphism was found to be associated with NIHL risk in the dominant model (GA/AA vs. GG) and allele model (G allele vs. A allele) including 1199 NIHL cases and 1211 controls. Comparing with the GG genotype, GA/AA genotype showed a decrease risk of NIHL. The G allele showed increasing risk comparing with A allele. In the subgroup analysis, the association was also found in steel factor workers, but not among chemical fiber company members. These results suggest that the potentially functional polymorphism rs3735715 may affect susceptibility to NIHL. As far as we know, this is the first comprehensive meta-analysis to estimate the association between the GRHL2 polymorphism and NIHL risk.

GRHL2, also known as Brother of Mammalian grainyhead (BOM) and Transcription Factor Cellular Promoter 2-Like 3 (TFCP2L3) is a member of GRHL transcription factor family which controls the development of multicellular epithelia by regulating cell junction formation and proliferation genes.^30^, ^31^ The junction proteins and ion channels play a critical role in the otic epithelial cells in inner-ear development and homeostasis maintenance. In zebrafish model, the mutant of GRHL2 shows inner-ear defects. Injecting wide-type human GRHL2 mRNA could rescue the defects.^32^ GRHL2 might influence the susceptibility to ARHI and DFNA28.^22^, ^24^ Just like ARHI and DFNA28, NIHL is one type of sensory impairment. Although they are not completely the same type hearing loss, some features are totally correspond, such as the hearing threshold in high frequency are most affected, and the sensorineural and progressive nature.^24^

In 2009, Konings et al.^33^ performed a large-scale association study in two independent noise-exposed populations to identify susceptibility genes for NIHL which did not find an association between GRHL2 and NIHL risk. The difference may be multifactorial, such as ethnic difference, different inclusion criteria, lifestyle, genetic and environmental factors. Our results about the rs3735715 polymorphism agreed with Yang's,^25^, ^34^ and Xu's^26^ studies while differed from Li's^27^ analysis. The reasons may be in Li's study,^27^ they chose workers from the chemical fiber company while others using steel factory subjects.^25^, ^26^, ^34^ Additionally, the definition of NIHL cases differed too. In Li's study,^27^ the workers with hearing threshold worse than 25 dB in high frequency were defined as NHIL. Otherwise in the other researches, the subjects with hearing threshold worse than 40 dB in high frequency were defined as NHIL.^25^, ^26^, ^34^ The negative result of Li's study about rs3735715 may also due to another reason. The effect of this polymorphism on the risk of NIHL might be too small to be detectable with the small sample size. The reason remains unclear, and studies using the same ethnic population are still needed to confirm these findings in the future.

An advantage of this meta-analysis was that numbers of subjects were pooled together from each independent study, which increased the statistical power significantly. Second, the quality of articles included in this analysis was satisfactory according to a consistent selection criterion. Third, based on this meta-analysis, functional study of rs3735715 in GRHL2 might be conducted to replicate these observations. There were some limitations of our meta-analysis. First, in the present study potential confounding factors (such as age, gender, exposure level, exposure time etc.) were not adjusted. Second, we only chose four papers written in English or Chinese. Although we estimated the publication bias using Egger's and Begg's test, we cannot ignore the possibility of bias. Third, we did not perform further evaluation of the potential interactions. The gene–gene or gene–environment interactions may modulate NIHL risk.^35^

## Conclusion

In conclusion, this meta-analysis found an association between the GRHL2 rs3735715 polymorphism and NIHL risk, suggesting GRHL2 might influence the susceptibility of NIHL. In the future, more extensive studies are still required to confirm these findings in different ethnic populations.

## Funding

This work was supported by the Humanities and Social Sciences of Ministry of Education Planning Fund of China (grant no. 16YJA840014).

## Conflicts of interest

The authors declare no conflicts of interest.
